# Uremic Pericarditis with Cardiac Tamponade in a Patient on Hemodialysis

**DOI:** 10.1155/2023/5099005

**Published:** 2023-11-06

**Authors:** Nismat Javed, Marcos Molina, Rabih Nasr, Gilda Diaz-Fuentes

**Affiliations:** ^1^Department of Internal Medicine, BronxCare Health System, Bronx, New York, USA; ^2^Department of Nephrology, BronxCare Health System, Bronx, New York, USA; ^3^Department of Pulmonology and Critical Care, BronxCare Health System, Bronx, New York, USA

## Abstract

Uremic pericardial effusion and pericarditis in end-stage kidney disease patients remain one of the causes responsible for high rates of morbidity and occasional mortality. While clinical presentation could be variable, clinicians should have a high index of suspicion for uremic pericarditis especially in patients who miss their dialysis sessions. We present a 77-year-old man with end-stage renal disease on dialysis diagnosed with pericarditis and large pericardial effusion complicated by cardiac tamponade and shock. He underwent urgent pericardiocentesis with clinical improvement. The course of the disease can be complicated by shock with multiorgan failure, particularly the liver. The presentation is relatively acute requiring a high level of suspicion, urgent diagnosis, and management to reduce mortality. As the geriatric population increases with associated comorbid conditions, it would be expected that patients undergoing dialysis would increase. Given the uncommon nature of the disease and how these patients have been managed by multiple specialties and care providers, it is important to consider dialysis-related complications in all patients with end-stage renal disease presenting with dyspnea.

## 1. Introduction

Dyspnea in patients on dialysis has a wide differential diagnosis from acute coronary syndrome, heart failure, fluid overload, pneumonia, infections, thromboembolic disease, and dialysis-related complications among others. In patients with end-stage renal disease (ESRD), pericarditis and pericardial effusion have been reported in up to 14% (range 2 to 21%) [1, 2]. Contrary to patients with non-ESRD, pericarditis in ESRD patients usually does not present with chest pains or typical ECG changes [3]. Pericardial effusion and pericarditis are responsible for 3–5% of deaths due to tamponade, fatal arrhythmia, and heart failure. [2]. Presentation of pericarditis in ESRD patients might be subtle; occasionally, they can develop hypotension and heart failure during dialysis, and this represents a diagnostic and management challenge.

We present a 77-year-old man with ESRD on dialysis diagnosed with pericarditis and large pericardial effusion leading to cardiac tamponade. We review the presentation, evaluation, and management of such patients.

## 2. Case Presentation

A 77-year-old man was hospitalized with one day of acute shortness of breath after missing 2 hemodialysis sessions, and he denied chest pain. He had history of hypertension, type 2 diabetes mellitus, hyperlipidemia, heart failure with preserved ejection fraction, ESRD on maintenance hemodialysis three times a week, benign prostatic hyperplasia, and chronic respiratory failure due to chronic obstructive airway disease on home oxygen. Family and social histories were not revealed. On admission, he was afebrile, with a heart rate of 79 beats/min, blood pressure of 110/52 mmHg, respiratory rate of 22/minute, and oxygen saturation of 100% on 4 L.

**Figure 1:**
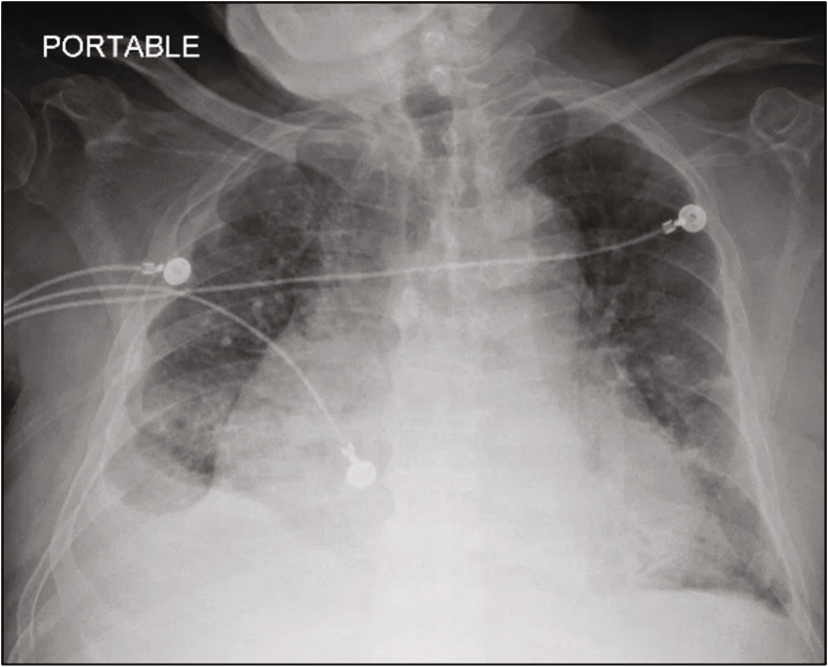
Chest X-ray showing pulmonary congestion, bilateral pleural effusion, cardiomegaly, and possible consolidation in the right lower lobe.

On examination, the patient was in mild distress with bilateral lung crackles and no leg edema or jugular venous distension. Electrocardiogram revealed atrial fibrillation and a heart rate of 75/minute. Initial chest X-ray suggested pulmonary congestion and possible consolidation (Figure 1).

He was started on antibiotics for suspected pneumonia and underwent emergency dialysis. He received metoprolol for atrial fibrillation, and he declined anticoagulation. Initial laboratory showed elevated blood urea nitrogen and serum creatinine and transaminitis. Repeated laboratory on day 2 of admission showed acute increase in transaminitis with alanine aminotransferase (ALT) of 2442 unit/L and aspartate aminotransferase (AST) levels of 2676 unit/L. Prior to the second session of dialysis on day 2 after admission, he developed hypotension. He was transferred to the ICU for suspected ischemic hepatopathy and shock; he was started on vasopressors. Echocardiography revealed an ejection fraction of 46.76% and large pericardial effusion with tamponade physiology; he underwent urgent pericardiocentesis by cardiology. Approximately 1 liter of hemorrhagic and exudative pericardial fluid was drained, and WBC in fluid was 6200/mm^3^. The microbiological testing on pericardial fluid returned negative.

Repeat dialysis was performed. The following echocardiogram showed resolution of pericardial effusion. Gastroenterology, rheumatologic, and infectious disease workups were all negative. Autoimmune workup was negative.

**Figure 2:**
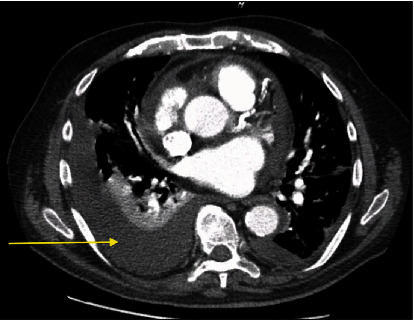
CT scan showing left-sided pleural effusion (yellow arrow).

A chest computed tomogram (CT) done on day 11 to evaluate persistent dyspnea revealed a left-sided pleural effusion and no pulmonary embolism or parenchymal disease (Figure 2).

A left-side thoracentesis was performed, and the pleural fluid analysis was consistent with transudative effusion which was attributed to heart failure and fluid overload.

The patient's clinical condition slowly improved, he completed the course of antibiotics, his transaminitis was corrected, and he was discharged home to continue dialysis as outpatient.

Summary of pertinent laboratory investigations is shown in Table 1.

He has been followed in the ambulatory clinics, and he is attending dialysis and remains stable without recurrence of pericardial effusion in repeated echocardiogram after 25 days of initial hospitalization.

## 3. Discussion

We presented a patient with ESRD on maintenance dialysis complicated with pericarditis and cardiac tamponade requiring pericardiocentesis. The prevalence of pericarditis in patients with ESRD varies from 2% to 21% [2, 4]. Dialysis-induced pericarditis has been reported in patients of varied age groups ranging from 50 to 90 years of age [4, 5] like our patient.

Pericardial effusion is a common finding in echocardiograms and can be present in approximately 6.5% of the general adult population, with higher incidence in high-risk populations. Pericardial effusion is classified as simple or complex, depending on the consistency of the pericardial fluid; majority of pericardial effusions are simple and small, usually asymptomatic and without significant physiologic alterations. They occur because of pericardial inflammation and increased microvascular permeability. A pericardial effusion can precipitate cardiac tamponade and, when untreated, can cause abrupt hemodynamic instability [6, 7].

Pericardial effusions are observed across a wide demographic and clinical spectrum, and patients presenting with significant pericardial effusion represent a diagnostic and management challenge. Risk factors for developing pericardial effusion include pericarditis secondary to infection, rheumatological diseases, postsurgical changes, renal pathologies, and malignancy [8].

Causes of cardiac tamponade in patients on hemodialysis include uremic pericarditis and dialysis-associated pericarditis. Uremic pericarditis is defined as pericarditis that occurs before or within eight weeks of initiating dialysis, and our patient has been on dialysis for 5 months and 24 days. Dialysis-related pericarditis is defined as pericarditis that occurs in patients who have been on dialysis for more than 8 weeks [9, 10].

Risk factors for the development of dialysis-associated pericarditis include accumulation of toxins, inadequate dialysis in stable patients, or relatively inadequate dialysis in patients with higher catabolic activity due to multiple comorbidities [3]. In our case, the patient had multiple poorly controlled comorbid conditions including diabetes, hypertension, COPD, heart failure, and anemia. Poor adherence to regular dialysis is another factor in our patient contributing to toxin accumulation.

Clinical presentation in dialysis-associated pericarditis is nonspecific with dyspnea, abdominal pain, hypoxemia, and occasionally fever or chest pain being reported; laboratory abnormalities are usually related to the renal disease [4, 5, 8]. Findings of leukocytosis, hypocalcemia, transaminitis, and elevated lactic acid, as observed in our patient, are relatively uncommon and not specific [4, 5, 8]. Chest imaging often shows cardiomegaly and pulmonary congestion patterns which suggest cardiac dysfunction [2–5, 11, 12].

Diagnosis requires a high index of suspicion; echocardiogram is an excellent diagnostic tool for pericardial effusion or tamponade in general. Echocardiogram could show dilated inferior vena cava, diastolic dysfunction, right ventricular collapse, and accumulation of fluid [4, 5].

Guidelines from the European Society of Cardiology for the management of renal failure-associated pericarditis include dialysis, pericardiocentesis or pericardial drainage, NSAIDs, corticosteroids, and colchicine. Management is based on the clinical presentation and hemodynamic stability of the patient [2, 8, 13].

In patients who are stable with small-sized effusions, frequent sessions of dialysis can alleviate the problem [6, 9, 14]. Trials of anti-inflammatory medications have been reported to be beneficial [9]. However, in our patient, due to hemodynamic instability and large effusion with tamponade, pericardiocentesis is the recommendation of choice [4, 9]. Analysis of pericardial fluid is needed to evaluate for other causes of pericardial effusion, especially infectious causes, tuberculosis, and malignancy where management will be modified.

Our patient had exudative hemorrhagic effusion with a negative workup for autoimmune, infectious, and malignant conditions and clinical and echocardiographic improvement with dialysis which suggests he had dialysis-induced pericarditis with tamponade.

Cardiac tamponade is usually observed in patients with *Staphylococcus aureus* bacteremia (87.5%) or influenza-induced myopericarditis (13.8% to 41.2%) [15, 16]. The mortality in these cases is generally high with rates as high as 99% [15, 16]. While the risk of developing pericardial tamponade has been observed to be lower for uremia in literature, the mortality rates can be relatively similar [17].

Prognosis depends on hemodynamic instability, adherence to maintenance dialysis, and optimization of comorbid conditions [4, 5, 8, 9, 14]. The presence of atrial fibrillation and cardiac tamponade in cases of dialysis-induced pericarditis was found to be associated with poor prognosis [2, 4]. Based on the reported data, our patient has all the risk factors for a poor prognosis.

## 4. Conclusions

Uremic pericarditis in ESRD patients warrants urgent intervention. Due to advances in dialysis and early institution of dialytic therapy, pericarditis became an uncommon presentation. However, clinicians should keep a high index of suspicion in the selected category of patients who misses frequently their dialysis sessions and have low dialysis clearance.

Optimization of all comorbid conditions, control of atrial fibrillation, and strict adherence to dialysis cannot be overemphasized to improve the prognosis of those patients. More studies are needed to understand the pathophysiology of the disease.

## Figures and Tables

**Table 1:** Pertinent laboratory investigations.

Blood work	Admission	Day # 2-ICU admission	Day # 13	At discharge, day # 22
Hemoglobin (g/dl)	7.7	7.3	9.5	8.6
WBC (uL)	9800	8900	9700	9500
Platelets (uL)	421000	403000	280000	300000
Sodium (mEq/L)	135	135	140	138
Potassium (mEq/L)	5.4	6.2	4.1	4.0
Calcium (mEq/L)	8.0	8.1	8.1	8.0
Chloride (mEq/L)	90	88	98	99
Glucose (mg/dl)	291	280	57	260
Bicarbonate (mEq/L)	22	16	28	26
Blood urea nitrogen (mg/dl)	76	72	40	32
Creatinine (mg/dl)	7	7.2	6	5.5
AST	344	2676	55	24
ALT	277	2442	197	29
ALP	181	205	172	177
Total bilirubin	0.3	0.6	0.7	0.6
Direct bilirubin	0.2	0.4	0.4	0.2

## Data Availability

Data can be made available on special request addressed to the corresponding author.

## Abbreviations

ESRD: End-stage renal disease
ECG: Electrocardiogram
WBC: White blood count.

## References

[1] Aghsaeifard Z., Firouzi R., Alizadeh R. (2022). Predisposing factors and uremic pericardial effusion among ESRD patients undergoing dialysis. *Annals of Medicine and Surgery*.

[2] Alpert M. A., Ravenscraft M. D. (2003). Pericardial involvement in end-stage renal disease. *The American Journal of the Medical Sciences*.

[3] Rehman K. A., Betancor J., Xu B. (2017). Uremic pericarditis, pericardial effusion, and constrictive pericarditis in end-stage renal disease: insights and pathophysiology. *Clinical Cardiology*.

[4] Abu-Abaa M., Hassan M., Mousa A., Arshad H., Shah S. (2023). Cardiac tamponade risk associated with anticoagulation for atrial fibrillation in dialysis-associated pericarditis: a case report. *Cureus*.

[5] Watase H., Oka K., Yamane F., Sano C., Ohta R. (2022). Hemodialysis-related pericarditis with cardiac tamponade. *Cureus*.

[6] Savage D. D., Garrison R. J., Brand F. (1983). Prevalence and correlates of posterior extra echocardiographic spaces in a free-living population-based sample (the Framingham study). *The American Journal of Cardiology*.

[7] Arntfield R. T., Millington S. J. (2012). Point of care cardiac ultrasound applications in the emergency department and intensive care unit - a review. *Current Cardiology Reviews*.

[8] Willner D. A., Goyal A., Grigorova Y., Kiel J. (2023). Pericardial Effusion. [Updated 2023 Feb 20]. *StatPearls*.

[9] Rosen R. J., Valeri A. M. (2023). Management of patients with kidney failure and pericarditis. *Clinical Journal of the American Society of Nephrology*.

[10] Park Y., Ko E. J., Chung B. H., Yang C. W. (2021). Kidney transplantation in highly sensitized recipients. *Kidney Research and Clinical Practice*.

[11] Gunukula S. R., Spodick D. H. (2001). Pericardial disease in renal patients. *Seminars in Nephrology*.

[12] Chugh S., Singh J., Kichloo A., Gupta S., Katchi T., Solanki S. (2021). Uremic- and dialysis-associated pericarditis. *Cardiology in Review*.

[13] Adler Y., Charron P., Imazio M. (2015). 2015 ESC guidelines for the diagnosis and management of pericardial diseases: the task force for the diagnosis and management of pericardial diseases of the European Society of Cardiology (ESC)endorsed by: the European Association for Cardio-Thoracic Surgery (EACTS). *European Heart Journal*.

[14] Peraino R. A. (1983). Pericardial effusion in patients treated with maintenance dialysis. *American Journal of Nephrology*.

[15] Radovanovic M., Petrovic M., Hanna R. D. (2022). Clinical presentation and management of methicillin-resistant Staphylococcus aureus pericarditis–systematic review. *Journal of Cardiovascular Development and Disease*.

[16] Radovanovic M., Petrovic M., Barsoum M. K. (2022). Influenza myopericarditis and pericarditis: a literature review. *Journal of Clinical Medicine*.

[17] Guild W. R., Bray G., Merrill J. P. (1957). Hemopericardium with cardiac tamponade in chronic uremia. *The New England Journal of Medicine*.

